# Factors Associated with Therapeutic Efficacy of Intravesical OnabotulinumtoxinA Injection for Overactive Bladder Syndrome

**DOI:** 10.1371/journal.pone.0147137

**Published:** 2016-01-29

**Authors:** Sheng-Mou Hsiao, Ho-Hsiung Lin, Hann-Chorng Kuo

**Affiliations:** 1 Department of Obstetrics and Gynecology, Far Eastern Memorial Hospital, Banqiao, New Taipei, Taiwan; 2 Department of Obstetrics and Gynecology, National Taiwan University Hospital, Taipei, Taiwan; 3 Department of Urology, Buddhist Tzu Chi General Hospital and Tzu Chi University, Hualien, Taiwan; Cardiff University, UNITED KINGDOM

## Abstract

**Objectives:**

To analyze the predictors of therapeutic efficacy after intravesical botulinum toxin A injection for overactive bladder syndrome (OAB) refractory to antimuscarinic therapy.

**Methods:**

All consecutively OAB patients, who visited the urologic outpatient clinics of a medical center and refractory to antimuscarinic treatment, were prospectively enrolled. All enrolled patients received intravesical injection of 100 U onabotulinumtoxinA (Botox). The Global Response Assessment (GRA) score ≥ 2 at 3 months after Botox injection was defined as a successful treatment, otherwise failed.

**Results:**

Overall, 89 patients received intravesical injection. Eighty patients, including 42 men and 38 women, had received follow-up at 3 months. The overall success rate was 63.8%. The global response assessment, urgency severity score, urgency, urgency urinary incontinence and frequency episodes, and functional bladder capacity improved after treatment. However, post-void residual volume (PVR) increased, and voiding efficiency (VE) decreased after treatment. Female gender (odds ratio = 3.75) was the only independent factor associated with the success. Female gender (coefficient = 0.74), low baseline overactive bladder symptoms score (coefficient = -0.12) and the presence of OAB-wet (coefficient = 0.79) were independent factors associated with therapeutic efficacy (i.e., GRA score). VE (odds ratio = 0.062) was the only predictor for a large PVR at 3 months. The optimum cutoff value of VE was <87% with the area under the ROC curve being 0.64 (sensitivity = 63.8%, specificity = 57.1%).

**Conclusions:**

The therapeutic effects of Botox can persist till 6 months after treatment. Female gender, low overactive bladder symptoms score and OAB-wet are associated better therapeutic efficacy, and low baseline VE is associated with large PVR. These findings can serve as an initial guide or assist in consultation regarding the treatment of OAB patients with Botox injection.

**Trial Registration:**

ClinicalTrials.gov NCT01657409

## Introduction

Overactive bladder syndrome (OAB) is characterized by urgency with or without urge incontinence, usually with frequency and nocturia [1]. Antimuscarinic agents are the first-line treatment and have a more than 70% of success rate [2]. Urothelial dysfunction and abnormalities of sensory receptor expression or transmitter release in the suburothelial nerves may contribute to OAB, which is refractory to antimuscarinics [3]. Intravesical treatment to inhibit abnormal receptor expression or transmitter release in the suburothelial space provides good therapeutic effects for the treatment of OAB [4].

Intravesical botulinum toxin A (BoNT-A) injection for OAB refractory to antimuscarinic therapy has emerged as a good treatment of choice. BoNT-A injection has both motor and sensory effects [5]. Despite a good therapeutic effect for BoNT-A injection was demonstrated in OAB, the resulting detrusor contractility impairment and large post-void residual volume (PVR) remained an important problem to be solved [6–8].

Base on the change on urodynamic parameters or quality of life assessment, the successful rate of BoNT-A injection for OAB ranged from 60% to 80% [6–13]. Makovey et al. reported that patients with previous histories of poor antimuscarinic efficacy had less therapeutic efficacy [14]. Sievert et al. reported that the number of prior antimuscarinics used of reason for their discontinuation did not affect the treatment response of BoNT-A injection [15]. However, the factor affecting the therapeutic effect of BoNT-A has not been reported, and this should be important for pre-treatment consultation. Thus, this post hoc analysis of a prospective study aims at investigate the factors that predict a better global outcome after intravesical BoNT-A injection in patients with OAB. Besides, we also investigated the serial changes of the subjective and objective outcome parameters after treatment.

## Materials and Methods

This study was a prospective investigation of 89 OAB patients who were treated with intravesical injection of 100 U of onabotulinumtoxinA (Botox, Allergan, Irvine, CA, USA) from August 2012 to June 2014. All patients received for the first time Botox injection. The intravesical injections of Botox were performed at 20 different sites of the bladder wall, excluding the trigone.

The inclusion criteria were urodynamically confirmed detrusor overactivity (DO) with or without urgency urinary incontinence (UUI) refractory to antimuscarinic treatment. The duration of antimuscarinic treatment should be at least 3 months. Patients had been treated with at least two different antimuscarinic agents and were still bothered by severe urgency or UUI of at least one episode per day. All patients were free of urinary tract infections, intrinsic sphincter deficiency, and neurogenic bladder on enrollment.

The institutional review board of the hospital approved the study (Tzu Chi General Hospital IRB: 101–39, S1 Protocol). This study is a post hoc analysis of the above study. All patients were informed about the possible adverse events after Botox injection and written informed consent was obtained from all patients before treatment. Twenty injections (5 U in 0.5 ml normal saline for each injection) in different sites were given to the patients.

Video-urodynamic studies were routinely performed for the diagnosis of DO, BOO, and intrinsic sphincter deficiency using Life-Tech urodynamics equipment (Stafford, Texas, USA). Women with BOO, detrusor underactivity (DU), and detrusor hyperactivity and inadequate contractility (DHIC) were also excluded from this study. BOO was defined as the radiologic evidence of bladder outlet narrowing plus a voiding pressure greater than 35 cm H2O and a maximum flow rate less than 15 mL/s or a voiding pressure greater than 40 cm H2O [16]. If the DO was associated with incomplete bladder emptying and PVR of more than 100 ml, DHIC was considered [17]. If patients did not have a voiding detrusor contractility of more than 10 cm H2O and needed to void by abdominal straining or were unable to void, DU was diagnosed [18].

Functional bladder capacity (FBC) was derived from 3-day voiding diary, which represents real-life bladder condition. Uroflowmetry for maximum flow rate, voided volume (VV) and PVR was performed at each visit. Total bladder capacity (TBC) was derived from the sum of voided volume (VV) and PVR. Voiding efficiency (VE) was defined as VV divided by TBC [19].

The urgency severity scale (USS) was measured using a modified version of the validated Indevus Urgency Severity Scale, which rated urgency severity by circling 0, 1, 2, or 3, defined as none, mild, moderate, and severe urgency, respectively [20]. In addition to the USS, which ranged from 0–3, we defined urgency incontinence as an UI score of 4. Besides, the overactive bladder symptom score (OABSS) [21] was also measured at each visits. OAB-wet was diagnosed while the presence of at least one episode of urgency incontinence in her 3-day bladder diary; otherwise, OAB-dry.

All patients were closely monitored monthly by personal interview after treatment for up to 6 months after the Botox injection. Any adverse events considered possibly related to Botox treatment were recorded.

The therapeutic efficacy was graded based on the Global Response Assessment (GRA, categorized into -3, -2, -1, 0, 1, 2, and 3, indicating markedly worse, moderately worse, mildly worse, no change, mildly improved, moderately improved, and markedly improved bladder symptoms, respectively). At 2 weeks, 1, 3 and 6 months after the Botox injection, patients were requested to report their subjective perception of the bladder condition which must be balanced by the improvement of OAB symptoms and any adverse effect emerged after Botox treatment. A GRA ≥ 2 at 3 months after Botox injection was defined as a successful treatment, otherwise failed [22].

The STATA software (Version 11.0; Stata Corp, College Station, TX, USA) was used for the statistical analyses. The Skillings-Mack test and Wilcoxon sign-rank test were used, as appropriate. The variables in the univariate regression analysis included gender, age, OABSS scores, USS scores, bladder diary and uroflowmetry variables, and the presence of OAB-wet. Multivariate backward stepwise regression analysis was performed using all variables from the univariate analysis. A *P* value of less than 0.05 was considered statistically significant. Receiver operating characteristic (ROC) curve analysis was performed to identify the optimal cutoff value for predicting the large PVR (i.e., defined as > 150 ml) after treatment. The optimal cutoff value was determined by the point on the ROC curve closest to the upper left-hand corner.

## Results

**Fig 1:**
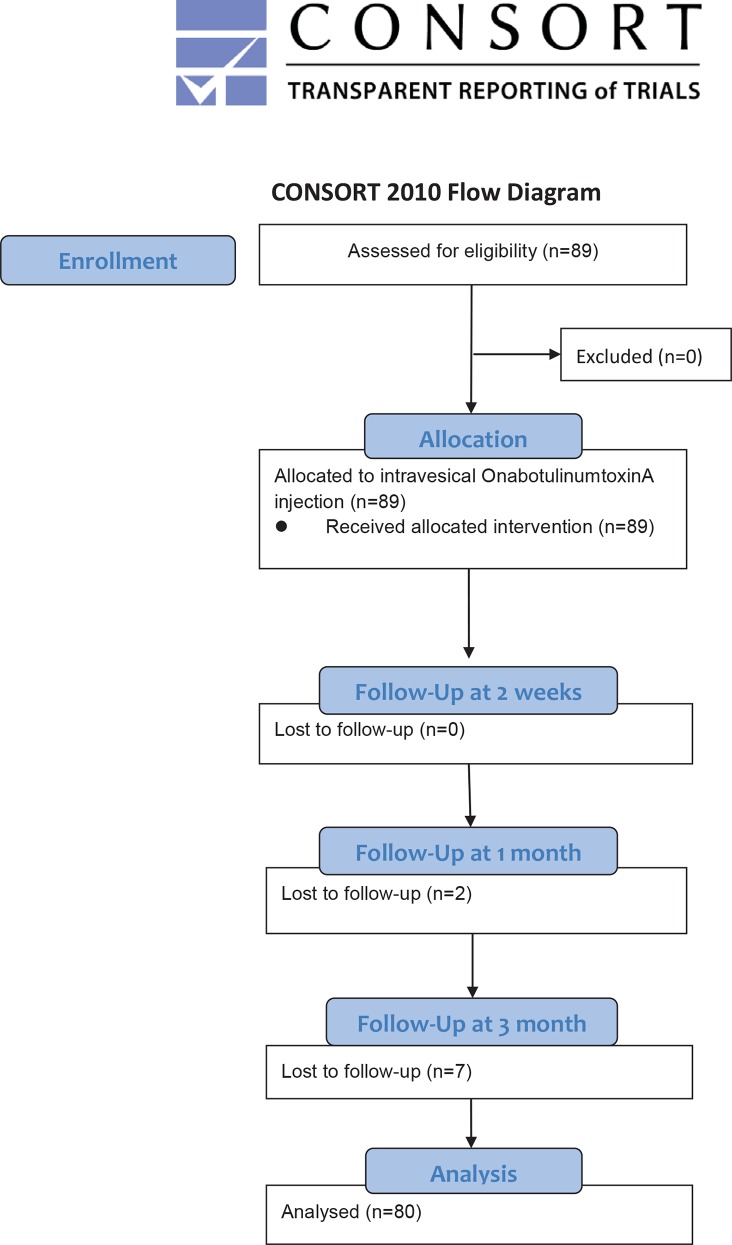
Flowchart of the participants with overactive bladder syndrome.

A total of 89 patients including 46 men and 43 women entered this prospective study (Table 1). The mean age was 64.7 ± 14.8 (ranged 23 to 89) years (S1 Dataset). Fig 1 listed the flow chart of patients follow-up.

**Table 1:** Baseline characteristics of both genders.

Variables	Male (n = 46)	Female (n = 43)	P-value^a^
Age (years)	70.5±11.9	58.5±15.2	<0.001
OABSS	11.0±2.5	12.4±2.1	0.01
USS	3.7±0.6	3.8±0.5	0.21
Urgency episodes (72 h)	26.9±13.8	33.5±17.1	0.09
UUI episodes (72 h)	6.5±10.5	10.2±11.8	0.009
Number of void (72 h)	36.2±13.6	40.8±15.4	0.26
VV(ml)	203±120	199±106	0.86
PVR (ml)	31±42	51±111	0.51
TBC (ml)	234±125	250±144	0.65
VE (%)	86±17	84±21	0.68
OAB-wet	19	43	<0.001

Values are given as mean ± standard deviation or number. OAB = overactive bladder syndrome; OABSS = overactive bladder symptoms score; PVR = postvoid residual volume; TBC = total bladder capacity; USS = urgency severity scale; UUI episodes = urgency urinary incontinence episodes on a 72 hour bladder diary; VE = voiding efficiency; VV = voided volume.

^a^ p values were calculated using the Wilcoxon ranksum test or Fisher exact test.

**Fig 2:**
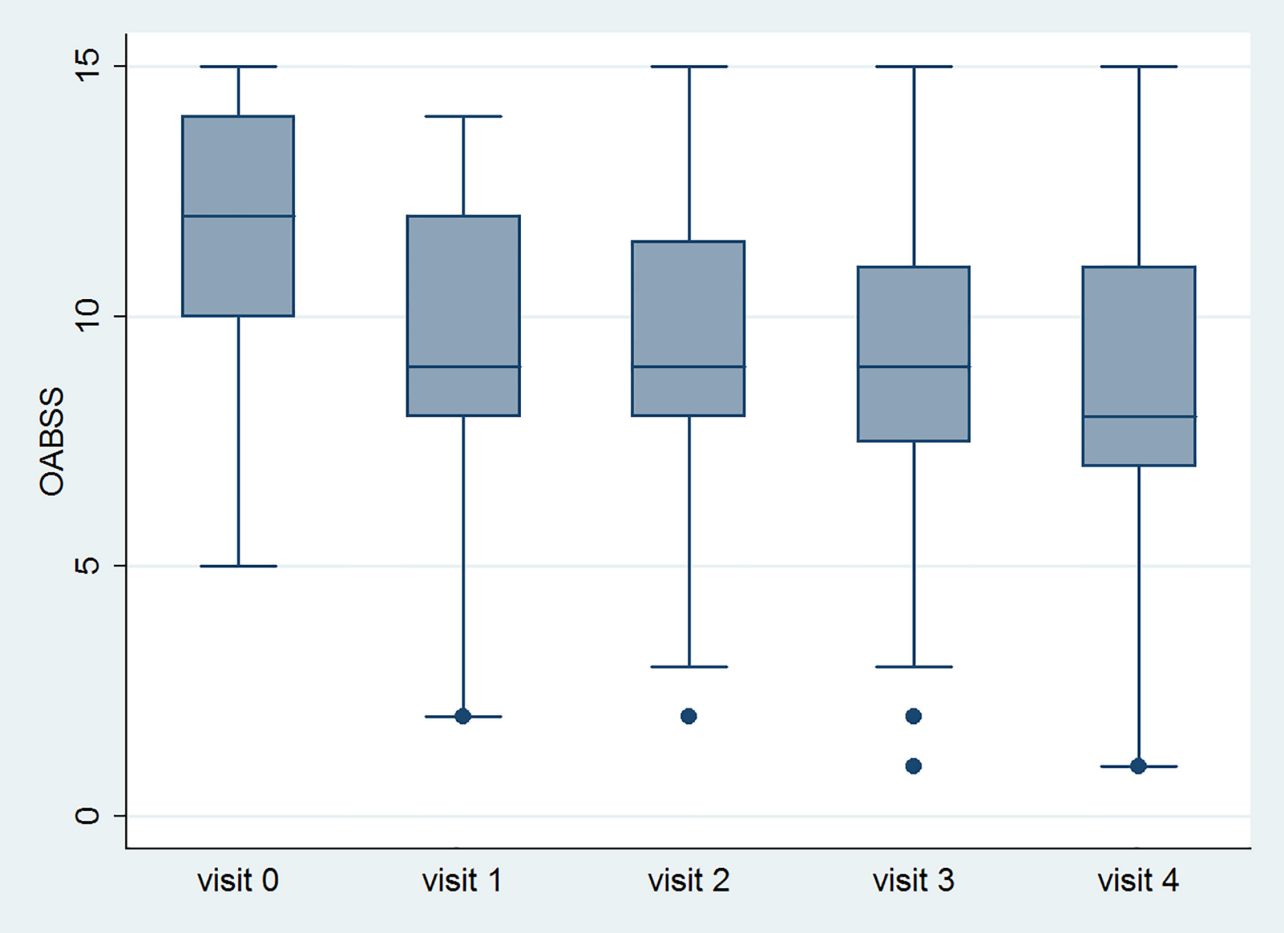
The changes of the Overactive Bladder Symptoms Scores after Botox injection with time.

**Fig 3:**
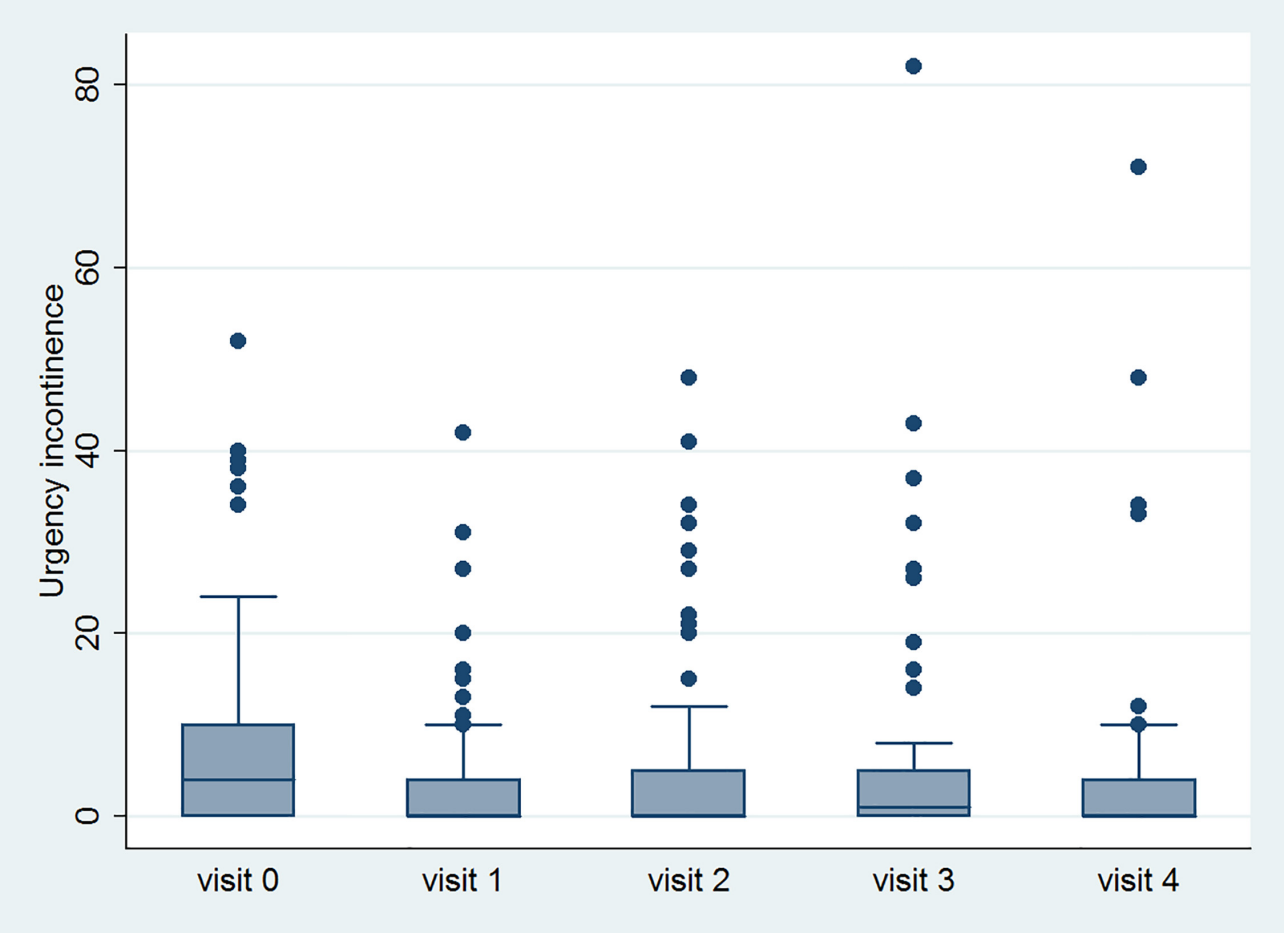
The changes of the episodes of urgency urinary incontinence after Botox injection with time.

**Fig 4:**
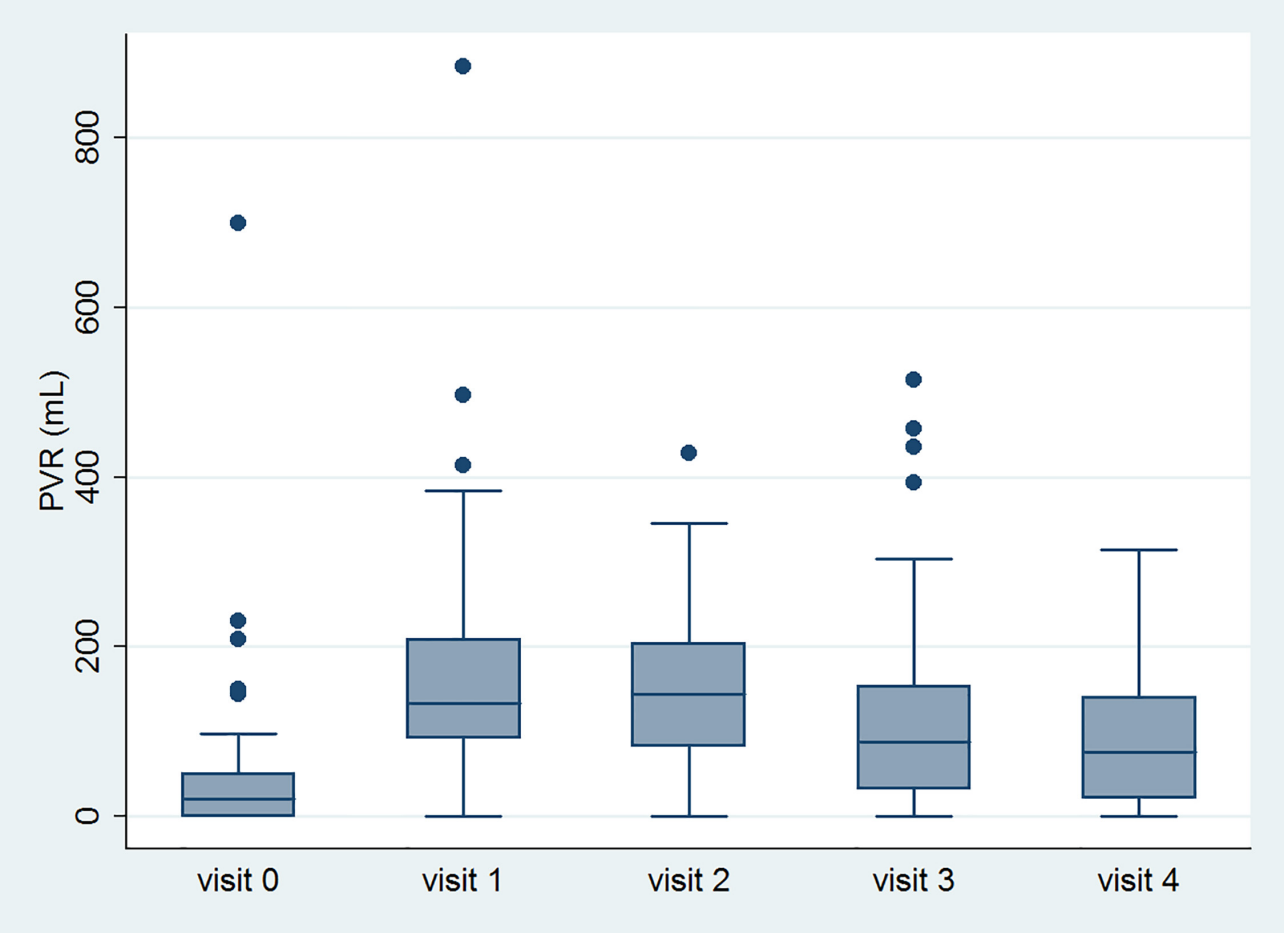
The changes of the postvoid residual volume after Botox injection with time.

**Fig 5:**
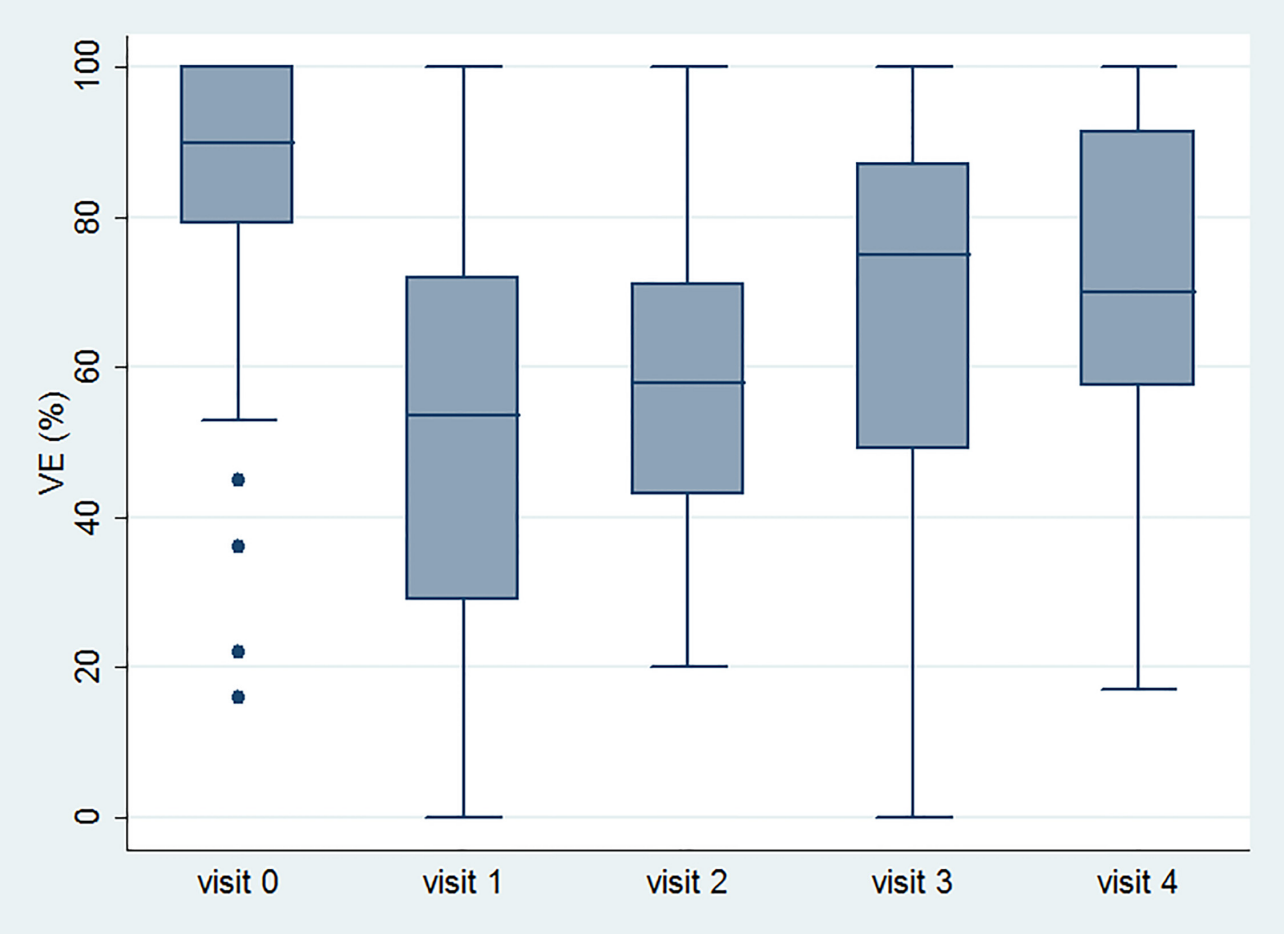
The changes of the voiding efficiency after Botox injection with time.

Table 2 lists the variable data at all time-points. GRA, OABSS, USS, urgency episodes, UUI episodes and number of void, TBC and number of OAB-wet improved after treatment. However, PVR increased and VE decreased after treatment. The significant changes of variables with time are shown in Figs 2, 3, 4 and 5.

**Table 2:** The follow-up data after treatment.

Variables	Baseline (a)	2 weeks (b)	1 month (c)	3 months (d)	6 months (e)	P value^a^	Post-hoc analysis^b^
Number	89	89	87	80	60	-	-
Age (years)	64.7±14.8	64.7±14.8	64.5±14.9	64.9±14.5	63.2±15.8	-	-
Gender							
Male	46	46	45	42	29	0.99	-
Female	43	43	40	38	31	-	
GRA	0±0	1.4±1.3	1.6±1.4	1.6±1.3	1.6±1.6	<0.001	a vs. b, c, d, e: all P <0.001
OABSS	11.7±2.4	9.3±3.2	9.3±3.3	9.1±3.4	8.4±3.4	<0.001	a vs. b, c, d, e: all P <0.001
USS	3.8±0.6	3.1±1.0	3.1±1.1	3.3±1.1	3.1±1.1	0.001	a vs. b, c, d, e: all P <0.001
Urgency episodes (72 h)	30.0±15.7	24.3±18.6	23.0±21.4	25.6±20.3	21.9±22.1	<0.001	a vs. b, d, e: all P <0.001; a vs. c, P = 0.02
UUI episodes (72 h)	8.2±11.2	4.1±8.5	5.1±10.1	5.5±12.5	5.0±12.5	<0.001	a vs. b, c, d, e: all P <0.001
Number of void (72 h)	38.3±14.5	37.9±16.4	35.6±15.1	34.9±16.1	32.9±18.1	<0.001	a vs. c, P = 0.02; a vs. d, e: P <0.01
VV(ml)	201±113	191±133	207±119	226±138	224±118	0.08	-
PVR (ml)	41±83	163±126	148±93	113±109	90±77	<0.001	a vs. b, c, d, e: all P <0.001
TBC (ml)	242±134	354±155	354±158	309±151	319±134	<0.001	a vs. b, c, d, e: all P <0.001
VE (%)	85±19	53±25	59±20	68±25	72±21	<0.001	a vs. b, c, d, e: all P <0.001
OAB-wet	62	31	37	39	27	<0.001	a vs. b, c, d, e: all P <0.001

Values are given as mean ± standard deviation or number. GRA = global response assessment; the other abbreviations are the same as Table 1.

^a^ p values were calculate using the Skillings-Mack test

^b^ p values of post hoc comparisons were performed using the Wilcoxon sign-rank test or McNemar test

Correlations between the GRA and the changes from baseline of all variables at 3 and 6 months were performed in Table 3. The GRA were well correlated to the changes of OABSS, urgency and UUI episodes at both 3 and 6 months, respectively, but not VV or TBC.

**Table 3:** Correlations between the GRA and the changes from baseline of all variables at 3 & 6 months.

Variables	Change from baseline at 3 months (n = 80)	Rho^a^	P-value	Change from baseline at 6 months (n = 60)	Rho^a^	P-value
OABSS	-2.5±3.5	-0.45	<0.001	-3.3±3.6	-0.45	0.0003
USS	-0.5±1.0	-0.33	0.003	-0.7±1.0	-0.22	0.11
Urgency episodes (72 h)	-2.5±19.4	-0.24	0.04	-8.4±23.3	-0.31	0.02
UUI episodes (72 h)	-1.7±11.5	-0.35	0.002	-2.6±12.1	-0.27	0.04
Number of void (72 h)	-1.7±14.2	-0.11	0.35	-7.1±17.0	-0.09	0.50
VV (ml)	22±117	0.16	0.16	6±115	0.05	0.69
PVR (ml)	67±107	0.10	0.40	41±100	0.04	0.76
TBC (ml)	62±158	0.05	0.67	55±160	0.03	0.82
VE (%)	-17±24	-0.04	0.69	-12±22	-0.02	0.88

Values are given as mean ± standard deviation or number (percentage). Abbreviations are the same as in Tables 1 & 2.

^a^ Spearman correlation coefficient.

Success rate was 63.8% (51/80; 95% confidence interval = 53.0% ~74.5%). Logistic regression analyses were performed to assess factors associated with the success (i.e., ≥2 of GRA at 3 months). Female gender, young age and the presence of OAB-wet were associated with the success in univariate analysis. However, female gender (odds ratio = 3.75) was the only independent factor associated with the success (Table 4).

**Table 4:** Univariate and multivariate logistic regression analyses of factors associated with therapeutic success at 3 months (n = 80).

	Therapeutic outcome		Univariate analysis		Multivariate analysis	
Variables	Success (n = 51)	Failure (n = 29)	OR (95% CI)	P-value	OR (95% CI)	P-value^a^
Female gender	30	8	3.75 (1.40~10.06)	0.009	3.75 (1.40~10.06)	0.009
Age (years)	62.1±15.6	69.9±11.1	0.96 (0.92~0.99)	0.03	-	-
OABSS	11.7±2.4	11.5±2.5	1.05 (0.87~1.27)	0.64	-	-
USS	3.8±0.5	3.7±0.6	1.53 (0.68~3.45)	0.30	-	-
Urgency episodes (72 h)	29.0±15.8	30.1±14.4	1.00 (0.97~1.03)	0.76	-	-
UUI episodes (72 h)	8.4±9.8	7.4±13.4	1.01 (0.97~1.05)	0.70	-	-
Number of void (72 h)	37.3±14.7	38.1±13.1	1.00 (0.96~1.03)	0.80	-	-
VV (ml)	208±118	194±104	1.001 (0.997~1.005)	0.59	-	-
PVR (ml)	40±98	48±64	0.999 (0.994~1.004)	0.69	-	-
TBC (ml)	248±148	242±109	1.000 (0.997~1.004)	0.25	-	-
VE (%)	87±16	81±24	1.01 (0.99~1.04)	0.23	-	-
OAB-wet	41	16	3.84 (1.32~11.1)	0.01	-	-

Values are given as mean ± standard deviation, number or odds ratio (95% confidence interval). CI = confidence interval; OR = odds ratio; other abbreviations as in Table 1.

^a^ p values were calculated using the backward stepwise multivariate logistic regression analysis with all variables from the univariate analysis.

Further analyses of factors associated with therapeutic efficacy (i.e., GRA score) at 3 months revealed that female gender, low baseline OABSS and the presence of OAB-wet were independent factors associated with therapeutic efficacy (Table 5).

**Table 5:** Univariate and multivariate linear regression analyses of factors associated with the GRA score at 3 months (n = 80).

	Univariate analysis		Multivariate analysis	
Variables	Coefficient (95% CI)	P value	Coefficient (95% CI)	P value^a^
Female gender	0.68 (0.15~1.23)	0.01	0.76 (0.25~1.27)	0.004
Age (years)	-0.02 (-0.04~-0.003)	0.09	-	-
OABSS	-0.07 (-0.18~0.05)	0.27	-0.12 (-0.24~-0.01)	0.03
USS	-0.04 (-0.56~0.47)	0.88	-	-
Urgency episodes (72 h)	-0.003 (-0.02~0.01)	0.67	-	-
UUI episodes (72 h)	0.001 (-0.02~0.02)	0.96	-	-
Number of void (72 h)	-0.01 (-0.03~0.01)	0.42	-	-
VV (ml)	0.001 (-0.001~0.004)	0.37	-	-
PVR (ml)	-0.001 (-0.004~0.002)	0.60	-	-
TBC (ml)	0.000 (-0.002~0.003)	0.68	-	-
VE (%)	0.017 (0.003~0.031)	0.02	-	-
OAB-wet	0.81 (0.24~1.38)	0.006	0.79 (0.19~1.38)	0.01

Values are given as coefficient (95% confidence interval). Abbreviations are the same as in Tables 1 & 2.

^a^ p values were calculated from the backward stepwise multivariate linear regression analysis with all variables from the univariate analysis.

**Fig 6:**
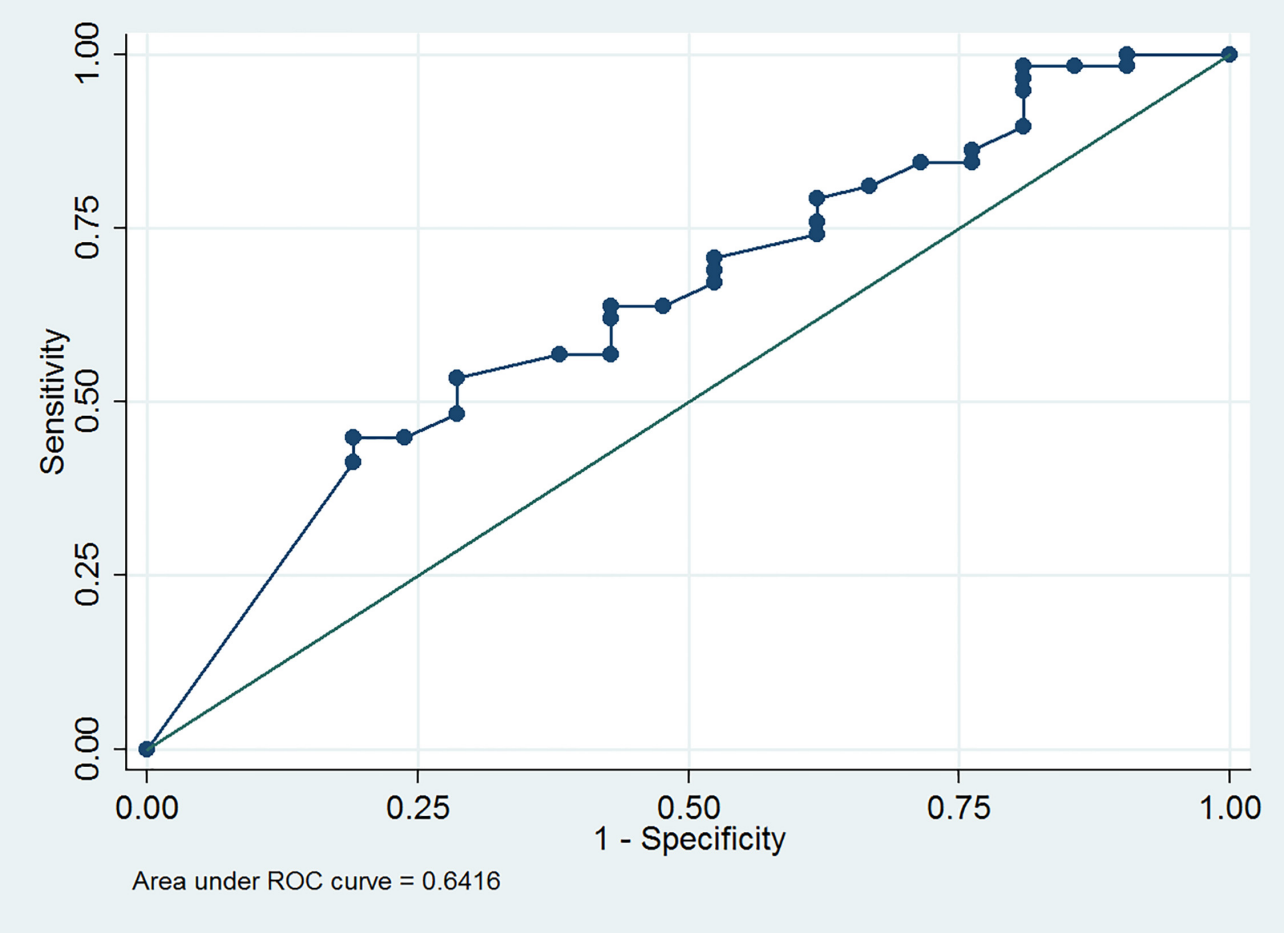
The area under the receiver operating characteristic (ROC) curve for voiding efficiency as a diagnostic test for the large postvoid residual volume.

Univariate and multivariate logistic regression analysis all revealed that VE (odds ratio = 0.973, 95% confidence interval = 0.948 to 0.997, P = 0.03, R^2^ = 0.05) is the only predictor for a large PVR at 3 months. The ROC analysis revealed that the optimum cutoff value of VE is <87 with the area under the ROC curve being 0.64 (95% CI = 0.50 to 0.78, sensitivity = 63.8%, specificity = 57.1%) (Fig 6).

Some points are not within the largest and smallest observations in the box plots, and may be considered as outliers. Visit 0 means baseline data. Visit 1, 2, 3 and 4 mean 2 weeks, 1 month, 3 month and 6 months after Botox injection, respectively.

## Discussion

This study revealed that intravesical Botox injection was significantly associated with a reduction of OABSS, USS, urgency and UUI episodes, number to void, and number of OAB-wet, but not VV, at all time-points and even at 6 months. A reduction of USS may be associated with sensory therapeutic effect, and an increase of bladder capacity may be correlated with motor therapeutic effect [5].

A previous study reported that the success rate of intravesical Botox injection was low among the patients who did not have sensory effects, and the therapeutic duration was significantly longer in patients who had sensory effects, compared to those with motor effects alone [5]. Thus, our current study supports the speculation that the therapeutic effect of intravesical Botox injection should be mainly related to sensory effect.

In addition, this study showed that the changes of OABSS, urgency and UUI after Botox injection were well correlated with the GRA at 3 and 6 months, but not the changes of VV and TBC, and these findings further support that sensory effect was more important in associated with therapeutic effect of Botox injection [5].

The PVR increased and VE decreased at all time-points. BoNT-A can cause paralysis of detrusor muscle by blocking acetylcholine release at the neuromuscular junction [23] and sensory impairment through reduction of purinergic receptor P2X3 and transient receptor potential vanilloid receptor subfamily 1 (TRPV1) expression on suburothelial sensory fibers [4]. Thus, it is impossible to achieve a successful therapeutic result for Botox injection purely via a sensory effect without compromising any detrusor contractility.

We found that female gender, low OABSS score and the presence of OAB-wet are associated with high therapeutic effects. To the best of our knowledge, there is no article mentioned about the above findings. Nonetheless, Makovey et al. reported that patients with poor antimuscarinic efficacy had less therapeutic efficacy of Botox injection [14]. A previous study revealed that female gender and high USS score were associated with high antimuscarinic efficacy [24]. USS score is derived from self-rating, and OAB-wet is derived from bladder diary. However, the correlation between USS score and the presence of OAB-wet is very high in current study (Spearman’s rho = 0.67, P<0.001). Thus, based on previous findings, the finding that female gender and the presence of OAB-wet were predictors of high therapeutic efficacy seems to be reasonable.

We also noted high OABSS was an independent factor for predicting low efficacy. However, its coefficient was low (i.e., -0.12). Thus, OABSS seems to be a poor predictor for assessing efficacy for Botox injection, compared with OAB-wet.

In addition, we found that low baseline VE is a predictor of large PVR at 3 months after Botox injection. Osborn et al. reported that preoperative PVR (odds ratio = 1.27) is associated with urine retention after Botox injection [25]. As mentioned, large PVR is associated low VE. Thus, our finding of low baseline VE as a predictor of large PVR seems reasonable. Despite baseline VE is not a good predictor owing to its low ROC area, patients with baseline VE < 0.87 can be informed about the possibility of large PVR after Botox injection.

This study has its limitations. First, the sample size is not large. However, we did get the factors affecting therapeutic efficacy via multivariate regression analysis. Second, a significant percentage (33%) of patients was lost to follow-up at 6 months; however, the rate of persistent antimuscarinic therapy was also low in patients treated for OAB. It had been reported to be 58% at 3 months and 35% at 12 months after the initiation of solifenacin treatment [26]. Thus, the low follow-up or persistent rates after treatment may be the distinct feature of OAB patients. Nonetheless, high percentage (90%) of patients was follow-up at 3 months, and this might make our analysis of therapeutic efficacy more reliable. Besides, OAB patient who did not demonstrate detrusor overactivity were excluded, thus our result may not generalize to OAB patients without detrusor overactivity.

## Conclusion

The therapeutic effects of Botox can persist till 6 months after treatment. Female gender, low OABSS and the presence of OAB-wet were associated better therapeutic efficacy. Low baseline VE is associated with large PVR. These findings could serve as an initial guide or assist in consultation regarding the treatment of OAB patients with Botox injection.

## Supporting Information

S1 CONSORT Checklist CONSORT 2010 checklist. (DOC)

S1 Dataset Raw data of this study. (XLS)

S1 Protocol The study record detail of the clinical trial. (PDF)

## References

[1] Abrams P, Abrams P, Cardozo L, Griffiths D, Rosier P, Ulmsten U, et al The standardisation of terminology of lower urinary tract function: Report from the Standardisation Sub-committee of the International Continence Society. Neurourol Urodyn. 2002; 21: 167–178. 11857671 10.1002/nau.10052

[2] Chapple CR. Muscarinic receptor antagonist in the treatment of overactive bladder. Urology. 2000; 55:.33–50. 10767450 10.1016/s0090-4295(99)00492-6

[3] Yiangou Y, Facer P, Ford A, Brady C, Wiseman O, Fowler CJ, et al Capsaicin receptor VR1 and ATP-gated ion channel P2X3 in human urinary bladder. BJU Int. 2001; 87: 774–779. 11412212 10.1046/j.1464-410x.2001.02190.x

[4] Apostolidis A, Popat R, Yiangou Y, Cockayne D, Ford AP, Davis JB, et al Decreased sensory receptors P2X3 and TRPV1 in suburothelial nerve fibers following intradetrusor injections of Botulinum toxin for human detrusor overactivity. J Urol. 2005; 174: 977–982. 16094018 10.1097/01.ju.0000169481.42259.54

[5] Kuo HC. Reduction of urgency severity is associated with long-term therapeutic effect after intravesical onabotulinumtoxin A injection for idiopathic detrusor overactivity. Neurourol Urodyn. 2011; 30: 1497–1502. 10.1002/nau.21132 21717501

[6] Kuo HC. Urodynamic evidence of effectiveness of botulinum A toxin injection in treatment of detrusor overactivity refractory to anticholinergic agents. Urology. 2004; 63: 868–872. 15134967 10.1016/j.urology.2003.12.007

[7] Sahai A, Khan MS, Dasgupta P. Efficacy of botulinum toxin-A for treating idiopathic detrusor overactivity: Results from a single center, randomized, double-blind, placebo controlled trial. J Urol. 2007; 177: 2231–2236. 17509328 10.1016/j.juro.2007.01.130

[8] Brubaker L, Richter HE, Visco A, Mahajan S, Nygaard I, Braun TM, et al Refractory idiopathic urge urinary incontinence and botulinum A injection. J Urol. 2008; 180: 217–222. 10.1016/j.juro.2008.03.028 18499184 PMC2597793

[9] Popat R, Apostolidis A, Kalsi V, Gonzales G, Fowler CJ, Dasgupta P. Comparison between the response of patients with idiopathic detrusor overactivity and neurogenic detrusor overactivity to the first intradetrusor injection of botulinum-A toxin. J Urol. 2005; 174: 984–989. 16094019 10.1097/01.ju.0000169480.43557.31

[10] Rajkumar GN, Small DR, Mustafa AW, Conn G. A prospective study to evaluate the safety, tolerability, efficacy and durability of response of intravesical injection of botulinum toxin type A into detrusor muscle in patients with refractory idiopathic detrusor overactivity. BJU Int. 2005; 96: 848–852. 16153215 10.1111/j.1464-410X.2005.05725.x

[11] Kessler TM, Danuser H, Schumacher M, Studer UE, Burkhard FC. Botulinum A toxin injections into the detrusor: An effective treatment in idiopathic and neurogenic detrusor overactivity? Neurourol Urodyn. 2005; 24: 231–236. 15747344 10.1002/nau.20105

[12] Werner M, Schmid DM, Schussler B. Efficacy of botulinum-A toxin in the treatment of detrusor overactivity incontinence: A prospective nonrandomized study. Am J Obstet Gynecol. 2005; 192: 1735–1740. 15902187 10.1016/j.ajog.2004.11.052

[13] Kuo HC. Clinical effects of suburothelial injection of botulinum A toxin in patients with non-neurogenic detrusor overactivity refractory to anticholinergics. Urology. 2005; 66: 94–98. 15992869 10.1016/j.urology.2005.02.002

[14] Makovey I, Davis T, Guralnick ML, O'Connor RC. Botulinum toxin outcomes for idiopathic overactive bladder stratified by indication: lack of anticholinergic efficacy versus intolerability. Neurourol Urodyn. 2011; 30: 1538–1540. 10.1002/nau.21150 21826718

[15] Sievert KD, Chapple C, Herschorn S, Joshi M, Zhou J, Nardo C, et al OnabotulinumtoxinA 100U provides significant improvements in overactive bladder symptoms in patients with urinary incontinence regardless of the number of anticholinergic therapies used or reason for inadequate management of overactive bladder. Int J Clin Pract. 2014; 68: 1246–1256. 10.1111/ijcp.12443 24754838 PMC4282287

[16] Kuo HC. Videourodynamic characteristics and lower urinary tract symptoms of female bladder outlet obstruction. Urology. 2005; 66: 1005–1009. 16286113 10.1016/j.urology.2005.05.047

[17] Resnick NM, Yalla SV. Detrusor hyperactivity with impaired contractile function. An unrecognized but common cause of incontinence in elderly patients. JAMA. 1987; 257: 3076–3081. 3586227 10.1001/jama.257.22.3076

[18] Kuo HC. Clinical symptoms are not reliable in the diagnosis of lower urinary tract dysfunction in women. J Formos Med Assoc. 2012; 111: 386–391. 10.1016/j.jfma.2011.05.014 22817816

[19] Abrams P. Bladder outlet obstruction index, bladder contractility index, and bladder voiding efficiency: three simple indices to define bladder voiding function. BJU Int. 1999; 84: 14–15. 10444116 10.1046/j.1464-410x.1999.00121.x

[20] Nixon A, Colman S, Sabounjian L, Sandage B, Schwiderski UE, Staskin DR, et al A validated patient reported measure of urinary urgency severity in overactive bladder for use in clinical trials. J Urol. 2005; 174: 604–607. 16006914 10.1097/01.ju.0000165461.38088.7b

[21] Homma Y, Yoshida M, Seki N, Yokoyama O, Kakizaki H, Gotoh M, et al Symptom assessment tool for overactive bladder syndrome—overactive bladder symptom score. Urology. 2006; 68: 318–323. 16904444 10.1016/j.urology.2006.02.042

[22] Jiang YH, Liu HT, Kuo HC. Decrease of urinary nerve growth factor but not brain-derived neurotrophic factor in patients with interstitial cystitis/bladder pain syndrome treated with hyaluronic acid. PLoS One. 2014; 9: e91609 10.1371/journal.pone.0091609 24614892 PMC3948883

[23] Simpson LL. Kinetic studies on the interaction between botulinum toxin type A and the cholinergic neuromuscular junction. J Pharmacol Exp Ther. 1980; 212: 16–21. 6243359

[24] Hsiao SM, Lin HH, Kuo HC. Factors associated with a better therapeutic effect of solifenacin in patients with overactive bladder syndrome. Neurourol Urodyn. 2014; 33: 331–334. 10.1002/nau.22394 23494586

[25] Osborn DJ, Kaufman MR, Mock S, Guan MJ, Dmochowski RR, Reynolds WS. Urinary retention rates after intravesical onabotulinumtoxinA injection for idiopathic overactive bladder in clinical practice and predictors of this outcome. Neurourol Urodyn. 2015; 34: 675–678. 10.1002/nau.22642 24975819 PMC4755310

[26] Wagg A, Compion G, Fahey A, et al Persistence with prescribed antimuscarinic therapy for overactive bladder: a UK experience. BJU Int. 2012; 110: 1767–1774. 10.1111/j.1464-410X.2012.11023.x 22409769

