# Supplementary material for: Factors Associated with Therapeutic Efficacy of Intravesical OnabotulinumtoxinA Injection for Overactive Bladder Syndrome
Source: PLoS One. 2016 Jan 29;11(1):e0147137. doi: 10.1371/journal.pone.0147137 (PMC4732812; doi:10.1371/journal.pone.0147137)
Supplement: S1 Protocol — (PDF) [file pone.0147137.s003.pdf]

ClinicalTrials.gov
A service of the U.S. National Institutes of Health

Example: "Heart attack" AND "Los Angeles"
Search for studies: Search
Advanced Search | Help | Studies by Topic | Glossary

Find Studies | About Clinical Studies | Submit Studies | Resources | About This Site

Home > Find Studies > Study Record Detail
Text Size

Different Injection Number of the Same Dose of Botulinum Toxin A on Overactive Bladder Syndrome

This study is currently recruiting participants. (see Contacts and Locations)

Verified April 2014 by Buddhist Tzu Chi General Hospital

Sponsor:
Buddhist Tzu Chi General Hospital

Information provided by (Responsible Party):
Hann-Chorng Kuo, Buddhist Tzu Chi General Hospital

ClinicalTrials.gov Identifier:
NCT01657409

First received: August 2, 2012
Last updated: April 3, 2014
Last verified: April 2014
History of Changes

Full Text View | Tabular View | No Study Results Posted | Disclaimer | How to Read a Study Record

▶ Purpose

Overactive bladder (OAB) is a symptom syndrome characterized by urgency frequency with or without urge urinary incontinence (UUI) that may affect the patients' quality of life. Current medical treatments are usually unsuccessful in completely eradicating urgency sensation. Intra-detrusor injection of botulinum toxin A (BoNT-A) modulates the release of neurotransmitters from sensory nerve endings and effectively modulates the inflammatory process mediated by nociceptive afferent nerve dysfunction. Satisfactory clinical results were achieved with intravesical BoNT-A injection, which increased bladder capacity and decreased urgency sensation in patients with neurogenic or idiopathic detrusor overactivity (NDO, IDO). Excellent results were achieved with injection of either 100 U or 200 U of BoNT-A. Episodes of frequency, urgency, and UUI were reduced, maximal cystometric capacity increased, maximal detrusor pressure (Pdet) decreased, and the quality of life index also improved significantly. However, post void residual (PVR) volume increased significantly and some patients required clean intermittent catheterization (CIC) to evacuate the PVR. Dose-related adverse events (AE) increased with increasing dose of BoNT-A. Therefore, adjustments of the BoNT-A dose and sites of injection might minimize the de novo AE and help to maintain success rates.

Condition	Intervention	Phase
Overactive Bladder	Drug: BoNT-A (10 injection)<br>Drug: BoNT-A (20 injection)<br>Drug: BoNT-A (40 injection)	Phase 2

Study Type: Interventional

Study Design: Allocation: Randomized
Endpoint Classification: Efficacy Study
Intervention Model: Parallel Assignment
Masking: Single Blind (Subject)
Primary Purpose: Treatment

Official Title: Comparison of the Therapeutic Effects of Different Injection Number of the Same Dose of Botulinum Toxin A on Overactive Bladder Syndrome

Resource links provided by NLM:

MedlinePlus related topics: Botox Overactive Bladder

Drug Information available for: Abobotulinumtoxina

U.S. FDA Resources

Further study details as provided by Buddhist Tzu Chi General Hospital:

Primary Outcome Measures:

- Global response assessment (GRA) of satisfaction by the patient [Time Frame: 2 weeks after initial treatment] [Designated as safety issue: Yes]

  GRA (-3, -2, -1, 0, +1, +2, +3) of satisfaction by the patient

  - GRA≥+1: Respond
  - GRA≤0: Non-respond

Secondary Outcome Measures:

- Voiding and urodynamic parameters [Time Frame: 2 weeks after the initial treatment day] [Designated as safety issue: Yes]
  - Overactive bladder symptom score (OABSS)
  - Urgency severity score (USS)
  - Urgency urinary incontinence (UUI)/3 days
  - Urodynamic parameters
  - Functional bladder capacity (FBC)
  - Maximum flow rate (Qmax)
  - Postvoid residual volume (PVR)

Estimated Enrollment:	90
Study Start Date:	August 2012
Estimated Study Completion Date:	June 2014
Estimated Primary Completion Date:	June 2014 (Final data collection date for primary outcome measure)

Arms	Assigned Interventions
Experimental: BoNT-A (10 injection)<br>100 U in 10ml, 1.0ml for each injection, totally 10 injections at bladder body	Drug: BoNT-A (10 injection)<br>BoNT-A 100 U 10 injections<br>Other Name: Botulinum Toxin A (Botox)
Experimental: BoNT-A (20 injections)<br>100 U in 10ml, 0.5ml for each injection, totally 20 injections at bladder body	Drug: BoNT-A (20 injection)<br>BoNT-A 100 U 20 injections<br>Other Name: Botulinum Toxin A (Botox)
Experimental: BoNT-A (40 injections)<br>100 U in 10ml, 0.25ml for each injection, totally 40 injections at bladder body	Drug: BoNT-A (40 injection)<br>BoNT-A 100 U 40 injections<br>Other Name: Botulinum Toxin A (Botox)

Detailed Description:

This study was designed as a single blind, randomized, parallel, actively controlled trial. The urodynamic DO confirmed patients were randomly assigned to receive injection of onabotulinumtoxinA 100 U (BoNT-A, Allergan, Irvine, California, USA), which was reconstituted to 10 ml with normal saline for suburothelial injections, in one of the three groups with the following injection number: (A) 100 U in 10ml injections, 1.0ml for each injection, totally 10 injections at bladder body (B) 100 U in 10ml, 0.5ml for each injection, totally 20 injections at bladder body, (C) 100 U in 10ml, 0.25ml for each injection, totally 40 injections at bladder body. Permuted block randomization was used for this trial. All treatments were evaluated at baseline and the primary end-point at 3 months.

The inclusion criteria were patients of either gender, aged 20 years or more, with urodynamic DO and at least one episode of urgency (urgency severity scale, USS≥2) or UUI per day as recorded in the 3-day voiding diary. Patients with neurogenic bladder, urodynamically confirmed bladder outlet obstruction, prior pelvic surgery, anti-incontinence surgery or urinary tract infection (UTI, white blood cell(WBC)>10/high power field (HPF) in urinalysis) were excluded. Informed consent was obtained from all patients before randomization. This study should be approved by the Institution Review Board and Ethics Committee of the hospital.

All patients had been managed with behavioral modification and treated with a certain number of antimuscarinics for more than 4 weeks before they were enrolled into this trial. Antimuscarinics was discontinued on the day of screening to wash out the remaining effect and obtaining a voiding diary that may reflect the true bladder condition.

The injection method for each patient was not recorded in the operation note and the study nurse who controlled the outcome measures was blinded to the treatment assignment. All procedures were performed transurethrally under intravenous general anesthesia in the operation room. Anticoagulant was discontinued 1 week prior to onabotulinumtoxinA treatment. The bladder volume was kept at 100-150 ml and the blood vessels were avoided during injections. An indwelling Foley catheter was placed in the bladder overnight and the patients were discharged the next morning. Broad-spectrum prophylactic antibiotics were given postoperatively for 3 days. Patients who developed acute urinary retention (AUR) or PVR volumes greater than 250 ml were advised to perform CIC periodically to evacuate their bladders. The patients were monitored at the outpatient clinic regularly for upto 24 months until symptoms returned to baseline levels.

Videourodynamic study was routinely performed at baseline, 3 and 6 months to measure urodynamic variables and detecting vesicoureteral reflux. The measured urodynamic variables included: maximum flow rate (Qmax), PVR, cystometric bladder capacity (CBC), detrusor pressure at Qmax (Pdet) and voiding efficiency (VE). The procedure and definition of videourodynamic study were in accordance of the recommendations of the International Continence Society.

▶ Eligibility

Ages Eligible for Study:	20 Years to 90 Years
Genders Eligible for Study:	Both
Accepts Healthy Volunteers:	No

Criteria

Inclusion Criteria:

- The inclusion criteria were patients of either gender, aged 20 years or more, with urodynamic DO and at least one episode of urgency (urgency severity scale, USS≥2) or UUI per day as recorded in the 3-day voiding diary. Patients with neurogenic bladder, urodynamically confirmed bladder outlet obstruction, prior pelvic surgery, anti-incontinence surgery or urinary tract infection (UTI, white blood cell (WBC) >10/high power field (HPF) in urinalysis) were excluded. Informed consent was obtained from all patients before randomization. This study should be approved by the Institution Review Board and Ethics Committee of the hospital.

Exclusion Criteria:

- Use of antimuscarinic agent and effective in treatment of lower urinary tract symptoms
- Patients with severe cardiopulmonary disease and such as congestive heart failure, arrhythmia, poorly controlled hypertension, not able to receive regular follow-up
- Patients with bladder outlet obstruction on enrollment
- Patients with postvoid residual > 150ml
- Patients with uncontrolled confirmed diagnosis of acute urinary tract infection
- Patients have laboratory abnormalities at screening including:
- Alanine aminotransferase (ALT)> 3 x upper limit of normal range
- Aspartate aminotransferase (AST)> 3 x upper limit of normal range
- Patients have abnormal serum creatinine level > 2 x upper limit of normal range
- Patients with any contraindication to be urethral catheterization during treatment
- Female patients who is pregnant, lactating, or with child-bearing potential without contraception.
- Myasthenia gravis, Eaton Lambert syndrome.
- Patients with any other serious disease considered by the investigator not suitable for general anesthesia or in the condition to enter the trial Patients participated investigational drug trial within 1 month before entering this study

Contacts and Locations

Choosing to participate in a study is an important personal decision. Talk with your doctor and family members or friends about deciding to join a study. To learn more about this study, you or your doctor may contact the study research staff using the Contacts provided below. For general information, see Learn About Clinical Studies.

Please refer to this study by its ClinicalTrials.gov identifier: NCT01657409

Contacts

Contact: Hann-Chorng Kuo, M.D. 886-3-8561825 ext 2113 hck@tzuchi.com.tw

Contact: Dong-Ling Tang, Miss 886-3-8561825 ext 2117 don_lin86@yahoo.com.tw

Locations

Taiwan

Buddhist Tzu Chi General Hospital **Recruiting**

Hualien, Taiwan, 970
Contact: Hann-Chorng Kuo, M.D. 886-3-8561825 ext 2113 hck@tzuchi.com.tw
Contact: Dong-Ling Tang, Miss 886-3-8561825 ext 2117 don_lin86@yahoo.com.tw

Sponsors and Collaborators

Buddhist Tzu Chi General Hospital

Investigators

Principal Investigator: Hann-Chorng Kuo, M.D. Department of Urology, Buddhist Tzu Chi General Hospital and Tzu Chi University

More Information

Publications:

Abrams P, Kelleher CJ, Kerr LA, Rogers RG. Overactive bladder significantly affects quality of life. Am J Manag Care. 2000 Jul;6(11 Suppl):S580-90. Review.

Irwin DE, Milsom I, Kopp Z, Abrams P, Cardozo L. Impact of overactive bladder symptoms on employment, social interactions and emotional well-being in six European countries. BJU Int. 2006 Jan;97(1):96-100.

Milsom I, Abrams P, Cardozo L, Roberts RG, Thüroff J, Wein AJ. How widespread are the symptoms of an overactive bladder and how are they managed? A population-based prevalence study. BJU Int. 2001 Jun;87(9):760-6. Erratum in: BJU Int 2001 Nov;88(7):807.

Chen GD, Lin TL, Hu SW, Chen YC, Lin LY. Prevalence and correlation of urinary incontinence and overactive bladder in Taiwanese women. Neurourol Urodyn. 2003;22(2):109-17.

Homma Y, Yamaguchi O, Hayashi K; Neurogenic Bladder Society Committee. An epidemiological survey of overactive bladder symptoms in Japan. BJU Int. 2005 Dec;96(9):1314-8.

Kullmann FA, Artim DE, Birder LA, de Groat WC. Activation of muscarinic receptors in rat bladder sensory pathways alters reflex bladder activity. J Neurosci. 2008 Feb 20;28(8):1977-87. doi: 10.1523/JNEUROSCI.4694-07.2008.

Mukerji G, Yiangou Y, Grogono J, Underwood J, Agarwal SK, Khullar V, Anand P. Localization of M2 and M3 muscarinic receptors in human bladder disorders and their clinical correlations. J Urol. 2006 Jul;176(1):367-73.

Ouslander JG. Management of overactive bladder. N Engl J Med. 2004 Feb 19;350(8):786-99. Review.

Dumoulin C, Hay-Smith J. Pelvic floor muscle training versus no treatment for urinary incontinence in women. A Cochrane systematic review. Eur J Phys Rehabil Med. 2008 Mar;44(1):47-63. Review.

Simpson LL. Kinetic studies on the interaction between botulinum toxin type A and the cholinergic neuromuscular junction. J Pharmacol Exp Ther. 1980 Jan;212(1):16-21.

Apostolidis A, Popat R, Yiangou Y, Cockayne D, Ford AP, Davis JB, Dasgupta P, Fowler CJ, Anand P. Decreased sensory receptors P2X3 and TRPV1 in suburothelial nerve fibers following intradetrusor injections of botulinum toxin for human detrusor overactivity. J Urol. 2005 Sep;174(3):977-82; discussion 982-3.

Aoki KR. Evidence for antinociceptive activity of botulinum toxin type A in pain management. Headache. 2003 Jul-Aug;43 Suppl 1:S9-15. Review.

Yiangou Y, Facer P, Ford A, Brady C, Wiseman O, Fowler CJ, Anand P. Capsaicin receptor VR1 and ATP-gated ion channel P2X3 in human urinary bladder. BJU Int. 2001 Jun;87(9):774-9.

Avelino A, Cruz F. TRPV1 (vanilloid receptor) in the urinary tract: expression, function and clinical applications. Naunyn Schmiedebergs Arch Pharmacol. 2006 Jul;373(4):287-99. Epub 2006 May 24. Review.

Popat R, Apostolidis A, Kalsi V, Gonzales G, Fowler CJ, Dasgupta P. A comparison between the response of patients with idiopathic detrusor overactivity and neurogenic detrusor overactivity to the first intradetrusor injection of botulinum-A toxin. J Urol. 2005 Sep;174(3):984-9.

Kuo HC. Urodynamic evidence of effectiveness of botulinum A toxin injection in treatment of detrusor overactivity refractory to anticholinergic agents. Urology. 2004 May;63(5):868-72.

Sahai A, Khan MS, Dasgupta P. Efficacy of botulinum toxin-A for treating idiopathic detrusor overactivity: results from a single center, randomized, double-blind, placebo controlled trial. J Urol. 2007 Jun;177(6):2231-6.

Brubaker L, Richter HE, Visco A, Mahajan S, Nygaard I, Braun TM, Barber MD, Menefee S, Schaffer J, Weber AM, Wei J; Pelvic Floor Disorders Network. Refractory idiopathic urge urinary incontinence and botulinum A injection. J Urol. 2008 Jul;180(1):217-22. doi: 10.1016/j.juro.2008.03.028. Epub 2008 May 21.

Reitz A, Stöhrer M, Kramer G, Del Popolo G, Chartier-Kastler E, Pannek J, Burgdörfer H, Göcking K, Madersbacher H, Schumacher S, Richter R, von Tobel J, Schurch B. European experience of 200 cases treated with botulinum-A toxin injections into the detrusor muscle for urinary incontinence due to neurogenic detrusor overactivity. Eur Urol. 2004 Apr;45(4):510-5.

Kessler TM, Danuser H, Schumacher M, Studer UE, Burkhard FC. Botulinum A toxin injections into the detrusor: an effective treatment in idiopathic and neurogenic detrusor overactivity? Neurourol Urodyn. 2005;24(3):231-6.

Rajkumar GN, Small DR, Mustafa AW, Conn G. A prospective study to evaluate the safety, tolerability, efficacy and durability of response of intravesical injection of botulinum toxin type A into detrusor muscle in patients with refractory idiopathic detrusor overactivity. BJU Int. 2005 Oct;96(6):848-52.

Schulte-Baukloh H, Weiss C, Stolze T, Stürzebecher B, Knispel HH. Botulinum-A toxin for treatment of overactive bladder without detrusor overactivity: urodynamic outcome and patient satisfaction. Urology. 2005 Jul;66(1):82-7.

Kuo HC. Clinical effects of suburothelial injection of botulinum A toxin on patients with nonneurogenic detrusor overactivity refractory to anticholinergics. Urology. 2005 Jul;66(1):94-8.

Ghei M, Maraj BH, Miller R, Nathan S, O'Sullivan C, Fowler CJ, Shah PJ, Malone-Lee J. Effects of botulinum toxin B on refractory detrusor overactivity: a randomized, double-blind, placebo controlled, crossover trial. J Urol. 2005 Nov;174(5):1873-7; discussion 1877.

Sahai A, Sangster P, Kalsi V, Khan MS, Fowler CJ, Dasgupta P. Assessment of urodynamic and detrusor contractility variables in patients with overactive bladder syndrome treated with botulinum toxin-A: is incomplete bladder emptying predictable? BJU Int. 2009 Mar;103(5):630-4. doi: 10.1111/j.1464-410X.2008.08076.x. Epub 2008 Oct 16.

White WM, Pickens RB, Doggweiler R, Klein FA. Short-term efficacy of botulinum toxin a for refractory overactive bladder in the elderly population. J Urol. 2008 Dec;180(6):2522-6. doi: 10.1016/j.juro.2008.08.030. Epub 2008 Oct 19.

Brady CM, Apostolidis AN, Harper M, Yiangou Y, Beckett A, Jacques TS, Freeman A, Scaravilli F, Fowler CJ, Anand P. Parallel changes in bladder suburothelial vanilloid receptor TRPV1 and pan-neuronal marker PGP9.5 immunoreactivity in patients with neurogenic detrusor overactivity after intravesical resiniferatoxin treatment. BJU Int. 2004 Apr;93(6):770-6.

Brady CM, Apostolidis A, Yiangou Y, Baecker PA, Ford AP, Freeman A, Jacques TS, Fowler CJ, Anand P. P2X3-immunoreactive nerve fibres in neurogenic detrusor overactivity and the effect of intravesical resiniferatoxin. Eur Urol. 2004 Aug;46(2):247-53.

Smet PJ, Moore KH, Jonavicius J. Distribution and colocalization of calcitonin gene-related peptide, tachykinins, and vasoactive intestinal peptide in normal and idiopathic unstable human urinary bladder. Lab Invest. 1997 Jul;77(1):37-49.

Yoshida M, Miyamae K, Iwashita H, Otani M, Inadome A. Management of detrusor dysfunction in the elderly: changes in acetylcholine and adenosine triphosphate release during aging. Urology. 2004 Mar;63(3 Suppl 1):17-23. Review.

Apostolidis A, Brady CM, Yiangou Y, Davis J, Fowler CJ, Anand P. Capsaicin receptor TRPV1 in urothelium of neurogenic human bladders and effect of intravesical resiniferatoxin. Urology. 2005 Feb;65(2):400-5.

Wiseman OJ, Fowler CJ, Landon DN. The role of the human bladder lamina propria myofibroblast. BJU Int. 2003 Jan;91(1):89-93.

Sui GP, Rothery S, Dupont E, Fry CH, Severs NJ. Gap junctions and connexin expression in human suburothelial interstitial cells. BJU Int. 2002 Jul;90(1):118-29.

Apostolidis A, Dasgupta P, Fowler CJ. Proposed mechanism for the efficacy of injected botulinum toxin in the treatment of human detrusor overactivity. Eur Urol. 2006 Apr;49(4):644-50. Epub 2006 Jan 4. Review.

Tyagi P, Wu PC, Chancellor M, Yoshimura N, Huang L. Recent advances in intravesical drug/gene delivery. Mol Pharm. 2006 Jul-Aug;3(4):369-79. Review.

Giannantoni A, Di Stasi SM, Chancellor MB, Costantini E, Porena M. New frontiers in intravesical therapies and drug delivery. Eur Urol. 2006 Dec;50(6):1183-93; discussion 1193. Epub 2006 Aug 30. Review.

Giannantoni A, Porena M, Costantini E, Zucchi A, Mearini L, Mearini E. Botulinum A toxin intravesical injection in patients with painful bladder syndrome: 1-year followup. J Urol. 2008 Mar;179(3):1031-4. doi: 10.1016/j.juro.2007.10.032. Epub 2008 Jan 18.

Kuo HC, Liu HT, Yang WC. Therapeutic effect of multiple resiniferatoxin intravesical instillations in patients with refractory detrusor overactivity: a randomized, double-blind, placebo controlled study. J Urol. 2006 Aug;176(2):641-5.

Birder LA. More than just a barrier: urothelium as a drug target for urinary bladder pain. Am J Physiol Renal Physiol. 2005 Sep;289(3):F489-95. Review.

Chuang YC, Yoshimura N, Huang CC, Chiang PH, Chancellor MB. Intravesical botulinum toxin a administration produces analgesia against acetic acid induced bladder pain responses in rats. J Urol. 2004 Oct;172(4 Pt 1):1529-32.

Liu HT, Chancellor MB, Kuo HC. Urinary nerve growth factor levels are elevated in patients with detrusor overactivity and decreased in responders to detrusor botulinum toxin-A injection. Eur Urol. 2009 Oct;56(4):700-6. doi: 10.1016/j.eururo.2008.04.037. Epub 2008 Apr 30.

Atiemo H, Wynes J, Chuo J, Nipkow L, Sklar GN, Chai TC. Effect of botulinum toxin on detrusor overactivity induced by intravesical adenosine triphosphate and capsaicin in a rat model. Urology. 2005 Mar;65(3):622-6.

Crowley SL, Worchel FF, Ash MJ. Self-report, peer-report, and teacher-report measures of childhood depression: an analysis by item. J Pers Assess. 1992 Aug;59(1):189-203.

Vemulakonda VM, Somogyi GT, Kiss S, Salas NA, Boone TB, Smith CP. Inhibitory effect of intravesically applied botulinum toxin A in chronic bladder inflammation. J Urol. 2005 Feb;173(2):621-4.

Fraser MO, Chuang YC, Tyagi P, Yokoyama T, Yoshimura N, Huang L, De Groat WC, Chancellor MB. Intravesical liposome administration--a novel treatment for hyperactive bladder in the rat. Urology. 2003 Mar;61(3):656-63.

Chuang YC, Tyagi P, Huang CC, Yoshimura N, Wu M, Kaufman J, Chancellor MB. Urodynamic and immunohistochemical evaluation of intravesical botulinum toxin A delivery using liposomes. J Urol. 2009 Aug;182(2):786-92. doi: 10.1016/j.juro.2009.03.083. Epub 2009 Jun 18.

Reimer K, Fleischer W, Brögmann B, Schreier H, Burkhard P, Lanzendörfer A, Gümbel H, Hoekstra H, Behrens-Baumann W. Povidone-iodine liposomes--an overview. Dermatology. 1997;195 Suppl 2:93-9. Review.

Egerdie RB, Reid G, Trachtenberg J. The effect of liposome encapsulated antineoplastic agents on transitional cell carcinoma in tissue culture. J Urol. 1989 Aug;142(2 Pt 1):390-8.

Tyagi P, Chancellor MB, Li Z, De Groat WC, Yoshimura N, Fraser MO, Huang L. Urodynamic and immunohistochemical evaluation of intravesical capsaicin delivery using thermosensitive hydrogel and liposomes. J Urol. 2004 Jan;171(1):483-9.

Chuang YC, Lee WC, Lee WC, Chiang PH. Intravesical liposome versus oral pentosan polysulfate for interstitial cystitis/painful bladder syndrome. J Urol. 2009 Oct;182(4):1393-400. doi: 10.1016/j.juro.2009.06.024. Epub 2009 Aug 15.

Liu HT, Kuo HC. Intravesical botulinum toxin A injections plus hydrodistension can reduce nerve growth factor production and control bladder pain in interstitial cystitis. Urology. 2007 Sep;70(3):463-8.

Truschel ST, Wang E, Ruiz WG, Leung SM, Rojas R, Lavelle J, Zeidel M, Stoffer D, Apodaca G. Stretch-regulated exocytosis/endocytosis in bladder umbrella cells. Mol Biol Cell. 2002 Mar;13(3):830-46.

Wang E, Truschel S, Apodaca G. Analysis of hydrostatic pressure-induced changes in umbrella cell surface area. Methods. 2003 Jul;30(3):207-17.

Cui M, Aoki KR. Botulinum toxin type A (BTX-A) reduces inflammatory pain in the rat formalin model. Cephalalgia 20:414-8, 2000.

Responsible Party:	Hann-Chorng Kuo, Department of Urology, Buddhist Tzu Chi General Hospital
ClinicalTrials.gov Identifier:	NCT01657409 History of Changes
Other Study ID Numbers:	TCGHUROL003
Study First Received:	August 2, 2012
Last Updated:	April 3, 2014
Health Authority:	Taiwan: Department of Health<br>Taiwan: Research Ethics Committee

Keywords provided by Buddhist Tzu Chi General Hospital:
botulinum toxin
detrusor overactivity

Additional relevant MeSH terms:
Urinary Bladder, Overactive
Lower Urinary Tract Symptoms
Signs and Symptoms
Urinary Bladder Diseases
Urologic Diseases
Urological Manifestations
Botulinum Toxins
Botulinum Toxins, Type A
Anti-Dyskinesia Agents
Central Nervous System Agents
Neuromuscular Agents
Peripheral Nervous System Agents
Pharmacologic Actions
Physiological Effects of Drugs
Therapeutic Uses

ClinicalTrials.gov processed this record on May 31, 2015
